# Observation of ventilation effects of I-gel™, Supreme™ and Ambu AuraOnce™ with respiratory dynamics monitoring in small children

**DOI:** 10.1007/s10877-016-9917-6

**Published:** 2016-08-04

**Authors:** Zhiqing Gu, Quanying Jin, Junjun Liu, Lianhua Chen

**Affiliations:** 1 Department of Anesthesiology, Shanghai Children’s Hospital, Shanghai Jiaotong University, Shanghai, China; 2 Department of Anesthesiology, Shanghai General Hospital, Shanghai Jiaotong University, Shanghai, China

**Keywords:** Respiratory dynamic monitoring, I-gel™, LMA-Supreme™, Ambu AuraOnce™, Children

## Abstract

The shortcomings of laryngeal mask airway (LMA™), such as upper airway obstruction and gastric distension or airway leakage, may limit its application in small children. The I-gel™ (I-gel), LMA-Supreme™ (LMA-S), and Ambu AuraOnce™ (Ambu) are three improvements upon these shortcomings. This study adopted respiratory dynamic monitoring to observe the ventilation parameters of the three laryngeal masks in small children. A total of 105 children were randomized into Ambu (n = 35), I-gel (n = 35), and LMA-S (n = 35) groups. Primary outcomes included leak pressure and respiratory dynamic data. Secondary outcomes included hemodynamic data and bispectral index values after induction (T_0_), time after successful laryngeal mask insertion (T_1_) and at three recording points every 10 min after insertion (T_2_, T_3_, and T_4_), as well as laryngeal mask related adverse reactions. The inspiratory/expiratory tidal volume per kilogram of body weight in the Ambu group was significantly different from those in the other groups (*P* < 0.05), while the leak pressure in the Ambu group was significantly lower (*P* < 0.05). At T_3_ and T_4_, the expiratory resistance values in the Ambu group were significantly lower than those in the LMA-S group (*P* < 0.05). We have shown that the three laryngeal masks provided secure ventilation in children <6 years of age by using continuous respiratory dynamic monitoring. We concluded that the I-gel presented a better sealing effect and fewer adverse reactions.

## Introduction

The laryngeal mask airway (LMA™), as one of the extraglottic devices (EGD), is favored in clinical anesthesia with the advantage of simple operation and less airway and cardiovascular reactions compared to tracheal intubation, as well as relatively steady airway compared to other EGDs including oropharyngeal airway, laryngeal tube airway and perilaryngeal airway, etc. The classical LMA for children is basically obtained by proportional size decreasing of the adult LMA without considering the characteristics of the anatomical structures of the pediatrics, such as a relatively large glossia, short neck, loose temporomandibular joint and high glottis. Thus it is thought to present higher risks of complications including airway leakage, displacement, insufficient ventilation, airway obstruction, as well as gastroesophageal regurgitation and aspiration [1]. Therefore, many anesthesiologists hesitate to use the LMA in children <6 years of age. The I-gel™ (I-gel), LMA-Supreme™ (LMA-S), and Ambu AuraOnce™ (Ambu) laryngeal masks are three improvements on the classical LMA [2–8]. The feature of Ambu is an arch in line with the axis of the oropharynx between its airway tube and cuff, which makes it difficult to displace, so it has been widely used for airway management in pediatric anesthesia in China. The I-gel and LMA-S are two types of laryngeal mask with gastroesophageal channel. Compared with the inflatable cuff of the LMA-S, the cuff of the I-gel is made by jelly-like elastomer gel, which makes it more plastic according to the shape of larynx.

Continuous airway monitoring (CAM) using a side stream spirometer (SSS) technique has been performed in real time to continuously observe respiratory dynamic parameters including the ventilation pressure, capacity, resistance, chest–lung compliance, pressure–volume loop, flow-volume loop, and respiratory work to facilitate a timely understanding of the mechanical state of the intraoperative lung and airway [9]. The reported detection rate of abnormal ventilation using CAM was significantly higher than that without CAM use during anesthesia in the same kind of surgeries [9–11]. Therefore, this study aimed to compare the ventilation effects of I-gel, LMA-S, and Ambu by using CAM technique, to verify the effectiveness and safety of applying these three laryngeal masks in the airway management of children <6 years of age under general anesthesia.

## Methods

### Research strategy

A total of 105 children aged 1–6 years, graded I according to American Society of Anesthesiologists (ASA) marking system, undergoing elective hypospadias repaired surgery were included. This study was approved by the ethics committee of Children’s Hospital, Shanghai Jiaotong University, and all parents provided written informed consent. The sample size was calculated based on pilot experiments, with a calculation formula: n = (Z_α/2_)^2^σ^2^/E^2^, 95 % confidence interval, <20 % tolerable error. The patients were divided into the Ambu, I-gel and LMA-S groups by sortition randomization method. Inclusion criteria were as follows: full-term birth; normal birth weight; no heart, lung, liver, kidney, or central nervous system function abnormalities; no history of gastroesophageal regurgitation; and no history of upper respiratory infection within 2 weeks before the surgery.

The children took no preoperative medicine before the anesthesia and were fasted routinely before they undergo elective operation. The electrocardiography, non-invasive blood pressure, pulse oxygen saturation (SpO_2_) (Datex-Ohmeda; GE), and bispectral index (BIS) (Vista™ BIS Monitoring System; Covidien) were monitored immediately after the children were sent to the operating room. The age, weight, height, heart rate (HR), mean arterial pressure (MAP), SpO_2_, and BIS of each child were recorded as baseline values. An intravenous catheter was inserted and Ringer’s solution was infused. For the anesthesia induction, the children received intravenous injections of sufentanil 0.2 μg/kg, atropine 0.01 mg/kg, midazolam 1 mg, and propofol 3 mg/kg. Meanwhile, oxygen was applied through a facemask connected to a respiratory monitor (Datex-Ohmeda Gas Exhaust E-CAiOV; GE).

After the eyelash reflex disappeared and the mandibular joint loosened, an appropriately size laryngeal mask according to each child’s weight and age was selected and inserted by the same anesthesiologist. The intracuff pressure of the laryngeal mask was monitored and adjusted to maintain a level within the green area (22–33 cm H_2_O) on the monitor (Manllinckrodt™ Hand Pressure Gauge; Covidien).

After the laryngeal mask insertion, the children were mechanical ventilated using the pressure-controlled mode (Datex-Ohmeda Aespire; GE) until the end of surgery. The initial parameters of pressure-controlled mode were as follows: the peak inspiratory pressure (PIP) was set at 15 cm H_2_O, respiratory rate was 20 breaths/min, inspiratory to expiratory ratio was 1:2, and positive end-expiratory pressure (PEEP) was set at zero. Meanwhile, a fiberoptic bronchoscopy (FOB; 2.2 mm; Olympus CLK-4) examination was used to determine laryngeal mask localization by a standard score described by Brimacombe [12]: Grade 4, only the glottis is visible; Grade 3, both the glottis and the posterior surface of the epiglottis are visible; Grade 2, both the glottis and the anterior surface of the epiglottis are visible; Grade 1, the glottis is invisible and normal ventilation is possible; and Grade 0, the glottis is invisible and normal ventilation is impossible. Cases with a FOB score of Grade 0 or with unobvious thoracoabdominal lifting, abnormal end-expiratory carbon dioxide (ETCO_2_) concentration, and flow-volume loop under the normal ventilation status after ventilator connection were identified as insertion failure for which re-insertion was required. Cases with three unsuccessful laryngeal mask insertions were changed to endotracheal intubation and were excluded from the study.

The leak pressure (LP) test was performed after the laryngeal mask location confirmation in supine position. Under the manual control ventilation mode, the APL valve (adjustable-pressure-limiting valve) in the breathing circuit of the anesthetic machine was closed, and the fresh gas flow was adjusted to 3 L/min to elevate the pressure in the breathing circuit until the airway pressure was stabilized, i.e. the LP. The testing was stopped if the airway pressure exceeded 40 cm H_2_O while unstable, while the LP was considered 40 cm H_2_O [13]. Gastric distension was identified by the auscultation of gurgling sounds during the inspiratory phase of mechanical ventilation as well as the comparison of abdominal perimeter pre- and postoperatively [14, 15].

Anesthesia was maintained by sevoflurane inhalation in 100 % oxygen in a 3 L/min fresh airflow combined with caudal block by 1 mL/kg of 0.3 % ropivacaine mesylate in all three groups of children, and no muscle relaxant was given. The inhalational concentration of sevoflurane was adjusted according to the BP, HR, and BIS value, while the PIP was adjusted based on the inspiratory tidal volume and ETCO_2_ value. The inhalation of the anesthetic gas was stopped 5 min before the end of surgery and the laryngeal mask was removed by the same anesthesiologist after spontaneous breathing was restored under deep anesthesia. The target of anesthesia management is: with a limited fluid infusion (8–10 mL kg^−1^ h^−1^), the MAP and HR were controlled within a range of baseline value ±20 %, the ETCO_2_ after laryngeal mask insertion was maintained at 30–40 mmHg, and the BIS value was stabilized at 40–60 during anesthesia.

### Outcome data

Main outcome measures were respiratory dynamic indexes including inspiratory tidal volume (VT_in_)/expiratory tidal volume (VT_ex_), PIP, and ETCO_2_. The inspiratory/expiratory tidal volume per kilogram of body weight [VT_(in/ex)_/kg], leakage fraction (LF) [(VT_in−_V_Tex_)/VT_in_] [16], and expiratory resistance (Re) [(plateau airway pressure-PEEP)/peak expiratory flow] were calculated [17].

Secondary outcome measures included hemodynamic data, bispectral index values and laryngeal mask related adverse reactions including hypoxemia (SpO_2_ < 90 %), gastric distension and regurgitation, frequent postoperative cough, hoarse cry, laryngospasm and bronchospasm.

Each measurement about hemodynamic data and BIS value was obtained immediately after induction of anesthesia (T_0_), when ETCO_2_ was stabilized after LMA insertion (T_1_), at 10 min (T_2_), 20 min (T_3_), and 30 min (T_4_) after LMA insertion. The LP and respiratory dynamic parameters were recorded at T_1_, T_2_, T_3_, and T_4_.

### Statistical analysis

All values are expressed as the mean ± standard deviation. Statistical analysis was conducted using SPSS software (IBM Corp. IBM SPSS Statistics for Windows, Version 20. Armonk, NY, USA). Data were analyzed by analysis of variance (ANOVA) with repeated measures. After the initial ANOVA, a series of stratified models were run to look for significant differences between groups at each time point using independent-samples *t* test or significant differences from baseline within each group using paired-samples *t* test. The enumeration data were tested using Fisher exact probability method. We considered a value of *P* < 0.05 to be statistically significant.

## Results

### General information of the patients

Among the enrolled 105 children, 10 were excluded due to laryngeal mask insertion failure indicated by large airway leakage, insufficient tidal volume and one of the following manifests: FOB score of Grade 0, unobvious thoracoabdominal lifting, abnormal end-expiratory carbon dioxide (ETCO2) concentration or flow-volume loop. Therefore, 95 were included in the study. The Ambu and I-gel groups each included 32 patients with successful insertion (91.43 %), while the LMA-S group included 31 patients with successful insertion (88.57 %). The general information of the patients is presented in Table 1. There was no significant difference between the groups with respect to age, weight and height. The size 2 laryngeal mask (suitable body weight 10–20 kg) was used in all patients. The insertion success rate and FOB score after successful insertion did not differ statistically among the three groups (*P* > 0.05, Table 1). The BIS value and hemodynamic indexes at each time point before and after LMA insertion did not show statistically significant differences among the three groups (*P* > 0.05) (Table 2).

**Table 1:** General information of the patients (mean ± SD)

	Ambu group	I-gel group	LMA-S group	*P* value
Number of cases with successful implantation	32	32	31	1.000
Age (m)	29.28±11.32	26.72 ± 12.16	31.16 ± 13.47	0.361
Weight (kg)	13.78 ± 2.55	13.95 ± 2.87	14.76 ± 2.92	0.252
Height (cm)	91.13 ± 9.93	88.84 ± 11.19	93.29 ± 10.49	0.337
FOB score (4/3/2/1/0)	0/16/15/1/0	0/17/15/0/0	0/16/13/2/0	0.830

Data were presented as mean ± SD

*P* value refers to comparison among three groups

**Table 2:** Hemodynamic data and BIS indexes at each time point by group (mean ± SD)

Time point	Item	Ambu group (n = 32)	I-gel group (n = 32)	LMA-S group (n = 31)	*P* value
T_0_	BIS	53.56 ± 2.29	54.06 ± 2.15	53.84 ± 2.35	0.640
	MAP (mmHg)	72.35 ± 6.42	71.89 ± 6.96	72.56 ± 7.51	0.645
	HR (beats/min)	116.56 ± 10.63	117.43 ± 9.74	118.54 ± 9.63	0.598
	SpO_2_ (%)	99.94 ± 0.45	99.64 ± 0.23	99.32 ± 0.47	0.958
T_1_	BIS	52.22 ± 2.47	52.66 ± 3.19	53.55 ± 3.64	0.299
	MAP (mmHg)	70.41 ± 5.32	71.51 ± 7.59	70.54 ± 8.69	0.687
	HR (beats/min)	112.45 ± 8.68	114.65 ± 7.69	115.48 ± 6.77	0.742
	SpO_2_ (%)	99.87 ± 0.14	99.34 ± 0.05	99.41 ± 0.16	0.933
T_2_	BIS	51.84 ± 3.32	51.72 ± 2.88	52.10 ± 3.73	0.900
	MAP (mmHg)	69.87 ± 6.53	68.94 ± 7.44	70.51 ± 6.79	0.218
	HR (beats/min)	112.49 ± 7.34	113.98 ± 5.69	115.31 ± 6.54	0.287
	SpO_2_ (%)	99.96 ± 0.03	99.89 ± 0.04	99.79 ± 0.15	0.932
T_3_	BIS	50.94 ± 3.59	51.31 ± 3.75	51.35 ± 3.52	0.880
	MAP (mmHg)	69.98 ± 7.51	69.14 ± 7.21	70.98 ± 5.67	0.269
	HR (beats/min)	112.96 ± 7.31	114.64 ± 6.62	114.97 ± 5.35	0.148
	SpO_2_ (%)	99.96 ± 0.03	99.57 ± 0.28	99.87 ± 0.31	0.965
T_4_	BIS	51.31 ± 3.80	52.72 ± 3.33	52.29 ± 3.98	0.344
	MAP (mmHg)	68.96 ± 5.36	68.74 ± 4.69	69.17 ± 6.71	0.203
	HR (beats/min)	112.07 ± 6.43	113.65 ± 7.61	114.86 ± 6.74	0.347
	SpO_2_ (%)	99.74 ± 0.04	99.91 ± 0.06	99.89 ± 0.05	0.943

Data were presented as mean ± SD

*P* value refers to comparison among the three groups

### Analysis of respiratory dynamic parameters

The LP in the Ambu group was significantly lower than that in the I-gel group (*P* < 0.05, Table 3). LF, VT_(in/ex)_, PIP, and ETCO_2_ did not show statistically significant differences among the three groups at any time points (*P* > 0.05). The VT_(in/ex)_/kg in the Ambu group was significantly different from those in the I-gel and LMA-S groups (*P* < 0.05). At the T_3_ and T_4_ time points, the Re in the Ambu group was significantly lower than that in the LMA-S group (*P* < 0.05) (Table 3).

**Table 3:** Pneumodynamic data at each time point by group

Time point	Item	Ambu group (n = 32)	I-gel group (n = 32)	LMA-S group (n = 31)	*P* value
T_1_	LP (cm H_2_O)	20.59 ± 4.90^ab^	24.38 ± 6.06^c^	23.71 ± 6.98	*P*_*a*_ = 0.008* *P*_*b*_ = 0.146 *P*_*C*_ = 0.595
	Inspiratory tidal volume (mL/kg)	11.22 ± 1.47^ab^	10.13 ± 1.17^c^	10.00 ± 1.42	*P*_*a*_ = 0.002* *P*_*b*_ = 0.001* *P*_*C*_ = 0.711
	Expiratory tidal volume (mL/kg)	11.13 ± 1.51^ab^	9.90 ± 1.2^c^	9.81 ± 1.34	*P*_*a*_ = 0.001* *P*_*b*_ = 0.000* *P*_*C*_ = 0.790
	RE (cm H_2_O/l/s)	55.67 ± 13.00	65.82 ± 19.00	62.97±20.92	0.071
T_2_	LP (cm H_2_O)	20.72 ± 4.56^ab^	24.38 ± 5.89^c^	23.55 ± 6.84	*P*_*a*_ = 0.010* *P*_*b*_ = 0.176 *P*_*C*_ = 0.352
	Inspiratory tidal volume (mL/kg)	10.50 ± 1.21^ab^	9.65 ± 0.94^c^	9.49 ± 1.17	*P*_*a*_ = 0.003* *P*_*b*_ = 0.001* *P*_*C*_ = 0.565
	Expiratory tidal volume (mL/kg)	10.41 ± 1.20^ab^	9.55 ± 0.97^c^	9.43 ± 1.14	*P*_*a*_ = 0.002* *P*_*b*_ = 0.001* *P*_*C*_ = 0.681
	RE (cm H_2_O/l/s)	60.29 ± 16.28	69.31 ± 20.70	68.01 ± 21.96	0.150
T_3_	LP (cm H_2_O)	20.72 ± 4.80^ab^	24.44 ± 5.79^c^	23.58 ± 6.79	*P*_*a*_ = 0.007* *P*_*b*_ = 0.183 *P*_*C*_ = 0.359
	Inspiratory tidal volume (mL/kg)	10.12 ± 1.16^ab^	9.31 ± 1.25^c^	9.02 ± 1.25	*P*_*a*_ = 0.010* *P*_*b*_ = 0.001* *P*_*C*_ = 0.348
	Expiratory tidal volume (mL/kg)	10.07 ± 1.12^ab^	9.28 ± 1.25^c^	8.91 ± 1.28	*P*_*a*_ = 0.011* *P*_*b*_ = 0.000* *P*_*C*_ = 0.225
	RE (cm H_2_O/l/s)	60.37 ± 13.98^ab^	69.19 ± 20.44^c^	71.96 ± 22.51	*P*_*a*_ = 0.071 *P*_*b*_ = 0.019* *P*_*C*_ = 0.570
T_4_	LP (cm H_2_O)	20.81 ± 4.69^ab^	24.41 ± 5.75^c^	23.55 ± 6.75	*P*_*a*_ = 0.008* *P*_*b*_ = 0.214 *P*_*C*_ = 0.363
	Inspiratory tidal volume (mL/kg)	10.03 ± 1.26^ab^	9.33 ± 1.48^c^	9.14 ± 1.36	*P*_*a*_ = 0.040* *P*_*b*_ = 0.011* *P*_*C*_ = 0.587
	Expiratory tidal volume (mL/kg)	10.04 ± 1.26^ab^	9.33 ± 1.48^c^	9.14 ± 1.34	*P*_*a*_ = 0.040* *P*_*b*_ = 0.011* *P*_*C*_ = 0.587
	RE (cm H_2_O/l/s)	60.79 ± 14.16^ab^	69.95 ± 19.86^c^	73.34 ± 22.71	*P*_*a*_ = 0.068 *P*_*b*_ = 0.011* *P*_*C*_ = 0.448

Data were presented as mean ± SD

*P* refers to comparison among three groups, *P*_*a*_ refers to comparison between the A group and the I group, *P*_*b*_ refers to the comparison between the A group and the S group, *P*_*c*_ refers to comparison between the I group and the S group

* Statistically significant with *P* < 0.05

### Adverse reactions related to LMA ventilation

No children in the three groups developed intra- or postoperative hypoxemia or suffered from regurgitation, aspiration, or laryngealspasm and bronchospasm after the laryngeal mask removal. Except for the incidence of gastric distension being remarkably higher in the Ambu group (five cases, 15.63 %; *P* < 0.05), the incidence of adverse reactions did not differ significantly among the three groups (Table 4).

**Table 4:** Adverse reactions by group

	Ambu group (n = 32)	I-gel group (n = 32)	LMA-S group (n = 31)	*P* value
Hypoxemia	0	0	0	
Gastric distention	5	0	0	0.010*
Postoperative cough	6	1	5	0.121
Secretions	3	1	2	0.693
Bloody fluid in laryngeal mask	1	0	1	0.771
Hoarse cry	3	1	1	0.614
Regurgitation and aspiration	0	0	0	
Laryngospasm and bronchospasm	0	0	0	

*P* refers to comparison among the three groups

* Statistically significant with *P* < 0.05

## Discussion

The laryngeal masks have become the commonly used airway management tools in general anesthesia. However, the classical LMA has the shortage of more frequency of displacement, inconvenient airway suction, and easily increased airway resistance due to airway obstruction by secretions when used in children. The airway pressure required to induce gastric distension during mechanical ventilation in children is reportedly lower in inappropriately located LMA compared with appropriately located LMA, which easily leads to increased intra-abdominal pressure, inhibited respiration, and an increased risk of gastroesophageal reflux [1]. Therefore, there have been some improvements, such as I-gel and LMA-S, with respect to these drawbacks of traditional LMA. However, to date, studies on these improved laryngeal mask have focused on clinical applications and complications; as such, detailed comparative data on respiratory dynamics are lacking.

In recent years, with the improvements in respiratory function monitoring technology, CAM using the SSS technique in routine anesthesia has become increasingly more common. CAM is reportedly able to detect 18 kinds of abnormalities of ventilation condition [9] and catheter position [10, 11]. Meanwhile, it is able to reflect the tidal volume, peak airway pressure, airway plateau pressure, PEEP, ETCO_2_, and other ventilation effect indexes in real time as well as enable understanding of the changes of expiratory resistance in mechanical ventilation through calculations of respiratory dynamics.

This study compared the parameters of respiratory dynamics and proved that these three laryngeal masks were able to provide effective ventilation to small children who do not use muscle relaxants under surgery, and the airway pressure that was required to achieve the tidal volume under the pressure-controlled ventilation mode did not cause adverse effects.

A comparison of expiratory resistance found that at the T_3_ and T_4_ time points, the change of expiratory resistance may be because the inner diameters of the airway tubes in the I-gel and Ambu are larger than that in the LMA-S [18, 19]. Although the increased expiratory resistance might lead to a reduced tidal volume and elevated peak airway pressure, it remained in a clinically acceptable range and no significant adverse effects occurred within 30 min. The outcomes from extended-length surgeries require further investigations.

LP and LF are the main indicators for assessing airway sealing and gas leakage in the entire ventilation process of applying supraglottic airway tools such as laryngeal mask, in which good LP and LF may ensure effective ventilation in the use of laryngeal mask in small children. By measuring LP and calculating LF, we found that although the I-gel does not contain a cuff and cannot achieve the purpose of sealing the airway though regulating the cuff pressure as with Ambu and LMA-S, the gel material of its cover enables the achievement of small amplitude shaping based on the children’s oropharyngeal structures to achieve an better sealing effect.

For the adverse reactions of the three groups, no obvious hemodynamic changes have been observed before and after laryngeal mask insertion, which suggesting that the three laryngeal masks create very small degrees of respiratory irritation. Meanwhile, the gastroesophageal channel structure of the I-gel and LMA-S that allows gastroesophageal suction can effectively prevent the occurrence of postoperative gastric inflation, thereby reducing the risk of gastroesophageal regurgitation and aspiration.

The cuffs of the LMA-S and Ambu are made of polyvinyl chloride (PVC). Some studies showed that PVC cuffs are more likely to induce a sore throat [20–22] in pediatric patients. In the current study, there were more cases of postoperative cough and hoarse cry in Ambu group compared to those in I-gel group, although no significant differences were found, which indicated that awareness of compression damage of the throat induced by over inflation of the cuff should be concerned. It has been reported that during nitrous oxide anesthesia, both cuff pressure and incidence of sore throat in the early postoperative period significantly increased in the classical LMA [23]. So it is regarded that the diffusion effect of nitrous oxide will lead the inflatable cuffs of laryngeal masks to a high inflation pressure. While the cover of the I-gel laryngeal mask is made of a kind of special thermoplastic elastomer and without an inflatable cuff. So it is unnecessary to worry about using nitrous oxide in I-gel laryngeal mask. These factors have become the dominant advantages of I-gel laryngeal mask in pediatric anesthesia.

## Conclusion

This study applied continuous airway monitoring technique to compare the ventilation effects of I-gel™, LMA-Supreme™ and Ambu AuraOnce™ in the children <6 years old. The three laryngeal masks had no obvious respiratory dynamic difference and were able to provide similar secure ventilation effects in small children with mechanical ventilation under general anesthesia without the use of a muscle relaxant. However, the I-gel™ had a better sealing effect, presented fewer postoperative adverse reactions. The I-gel™ presents some superiority over the other two types.
